# Modular eFAST tissue phantom for AI-based ultrasound triage

**DOI:** 10.1186/s12938-025-01448-8

**Published:** 2025-10-09

**Authors:** Isiah Mejia, Sofia I. Hernandez Torres, Carlos Bedolla, Rachel Gathright, Theodore Winter, Krysta-Lynn Amezcua, Eric J. Snider

**Affiliations:** Organ Support & Automation Technologies Group, U.S. Army Institute of Surgical Research, JBSA Fort Sam Houston, San Antonio, TX 78234 USA

## Abstract

Ultrasound (US) imaging is the primary choice for diagnosing and triaging patients in the battlefield as well as emergency medicine due to ease of portability and low-power requirements. Interpretation and acquisition of ultrasound images can be challenging and requires personnel with specialized training. Incorporating artificial intelligence (AI) can enhance the imaging process while improving diagnostic accuracy. To accomplish this goal, we have developed a full torso tissue-mimicking phantom for simulating US image capture at each site of the extended-focused assessment with sonography for trauma (eFAST) exam and is suitable for developing AI guidance and classification models. The US images taken from the phantom were used to train AI models for detection of specific anatomical features and injury state diagnosis. The tissue-mimicking phantom successfully simulated full thoracic motion as well as modular injuries at each scan site. AI models trained from the tissue phantom were able to achieve IOU’s greater than 0.80 and accuracy of 71.5% on blind inferences. In summary, the tissue mimicking phantom is a reliable tool for acquiring eFAST images for training AI models. Furthermore, the tissue phantom could be implemented for training personnel on ultrasound examination techniques as well as developing image acquisition automation techniques.

## Introduction

Ultrasound (US) imaging is a powerful tool used for medical diagnosis in both combat casualty care and emergency medicine due to its low cost and portability [1]. One of its most common uses is the extended-focused assessment with sonography for trauma (eFAST) exam [2]. The eFAST exam focuses on scanning specific anatomical regions to identify free fluid or air as an indicator of injury in the thorax or abdomen. This use of US enables the detection of abdominal hemorrhage (AH), pneumothorax (PTX), and hemothorax (HTX) injuries.

Although eFAST exams are essential for emergency diagnosis, they can require extensive training to appropriately position the US transducer and interpret the US images. Thus, skilled medical professionals are required to successfully conduct an eFAST exam and accurately interpret the US images. Necessary medical training for US imaging is not standard to military medics or civilian emergency medical technicians, limiting its utility in the pre-hospital environment [3]. To accomplish the goal of making US imaging viable in battlefield settings, the integration of artificial intelligence (AI) can enhance eFAST examination specifically through providing (i) guidance to properly image scan sites through anatomical recognition and (ii) automating image interpretation and diagnoses from captured images.

AI has been used in several fields in the healthcare industry. For medical imaging, AI has been previously used to diagnose diseases or abnormalities using magnetic resonance imaging [4], X-ray [5, 6], and computed tomography [7, 8]. AI has proven to be useful in reducing the time needed to detect and interpret abnormalities as well as improving the identification of features that are easily missed by the human eye [9]. In addition, closed-loop systems have been successful in stabilizing patients by using control systems to monitor fluids or drug administration [10, 11]. The combination of robotics and AI has been used to automate image acquisition and injury diagnosis during eFAST examinations in swine subjects [12]. For eFAST exams, object detection models can be used to automate US image acquisition by guiding US scanning to the optimal imaging site, allowing classification models to then automate injury diagnosis.

In order to develop deep learning AI models for medical imaging applications, large data sets are required that contain images of the relevant anatomy both with and without injury. Additionally, the AI models need images at various angles, which would not be appropriate during emergency eFAST examination procedures with an injured patient. Similarly, capturing this data in an animal model yields other time and cost challenges that fail to serve as a practical substitute to human studies. Thus, tissue-mimicking phantoms function as an alternative. There are multiple commercially available FAST and eFAST trainers with varying adjustable injury states and image quality capabilities. For example, the SonoSkin Trainer utilizes a life-like cover that can be scanned with a mock ultrasound probe and pre-programmed images are shown [13]. This trainer is very limited for AI development as there is no variance in the images, and the software does not account for varying probe angles during an eFAST exam. Other US compliant phantoms have been developed to overcome this limitation, such as the Blue Phantom FAST trainer which is made-up of ultrasound compliant materials and can simulate hemorrhage by injecting fluid around the heart, spleen, bladder and liver scan sites [14]. However, negative injury scans are not able to be collected for model training due to imaging artifacts left after fluid removal. Additionally, the phantom lacks a thoracic cavity with anatomically relevant features making diagnostic data collection for thoracic-related injuries impossible.

Previously, we developed a full body US compliant phantom with organs placed in the proper anatomical thoracic and abdominal eFAST scan sites that was designed to have both negative and positive injuries at each scan point except for the pericardial site [15]. Lung motion during breathing was simulated by using a rotating actuator to rotate a foam piece in the intercostal space. While the phantom design was successful at being able to conduct an eFAST examination, it was challenging to create variable injury states. Additionally, the breathing mechanism was only able to simulate lung motion at a single intercostal space.

To overcome these limitations, we developed an enhanced eFAST phantom called the Artificially Designed Anatomical Model (ADAM). ADAM is an US compliant tissue phantom designed to simulate lung motion and create modular injuries, including HTX, PTX, and AH. These modular injuries are arranged in the anatomically correct scan sites for eFAST examinations. For thoracic scan points, the chest cavity was made hollow, and breathing was simulated using the Expiration Via Extension (EVE) system, enabling lung motion along the entire thoracic region of the phantom. The tissue phantom can also serve as a training tool for personnel to gain experience in eFAST examinations. Specifically, this tissue phantom model can help with proper US probe placement by ensuring the target organ(s) are in focus. ADAM’s unique features offer valuable training opportunities, such as being exposed to injuries of varying severities and image variance which can educate novices in diagnosis.

## Results

**Fig. 1:**
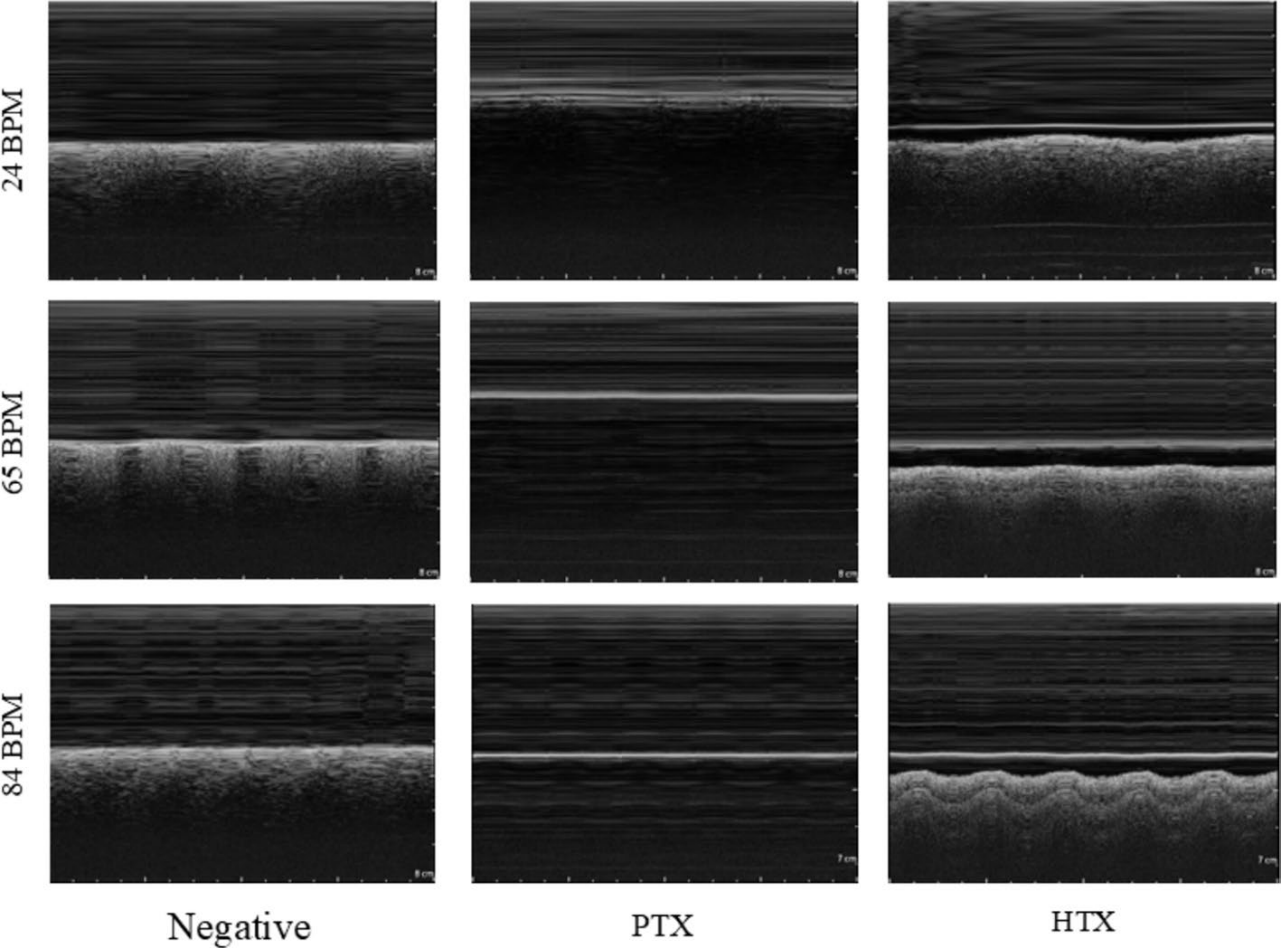
Representative M-mode ultrasound images for Negative, PTX, and HTX thoracic conditions at different breathing rates achieved by the EVE system for simulating lung motion (described in methods)

The EVE system successfully simulated breathing, enabling the imaging of physiologically representative breathing at multiple thoracic scan sites. Due to limitations of the DC motor controller, simulated breathing rates ranged from 24 to 84 breaths per minute (BPM), faster than typical breathing rates. Captured US motion mode(M-mode) images for both negative and positive PTX and HTX injuries at various BPMs can be seen in Fig. 1.

**Fig. 2:**
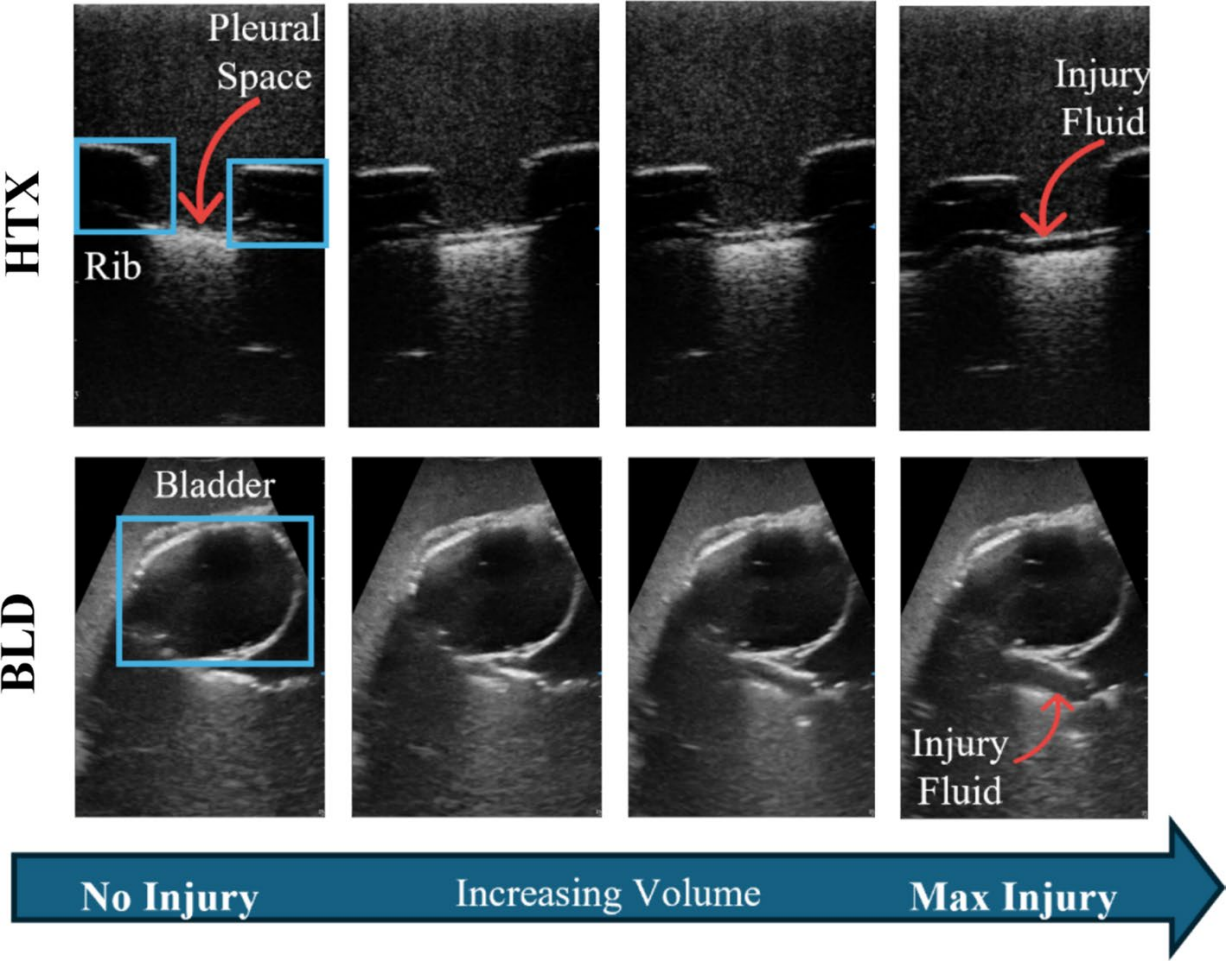
Annotated representative B-mode ultrasound images for the HTX and BLD scan points showing increasing injury volume. Images go from no injury to a full injury from left to right

The mechanism used to simulate injuries successfully modeled injury progression. Empty US sleeve pouches were initially placed at each scan site, and US images of negative injury states were collected. The injury severity was then progressed by filling the sleeves with fluid or air, simulating varying levels of injury. US images were then captured at different severities and scans sites, as shown in Fig. 2. As injury sizes increased in volume, the fluid became more pronounced in the US image in the pleural space for PTX and HTX injuries as well as around anatomical features at each abdominal site.

**Fig. 3:**
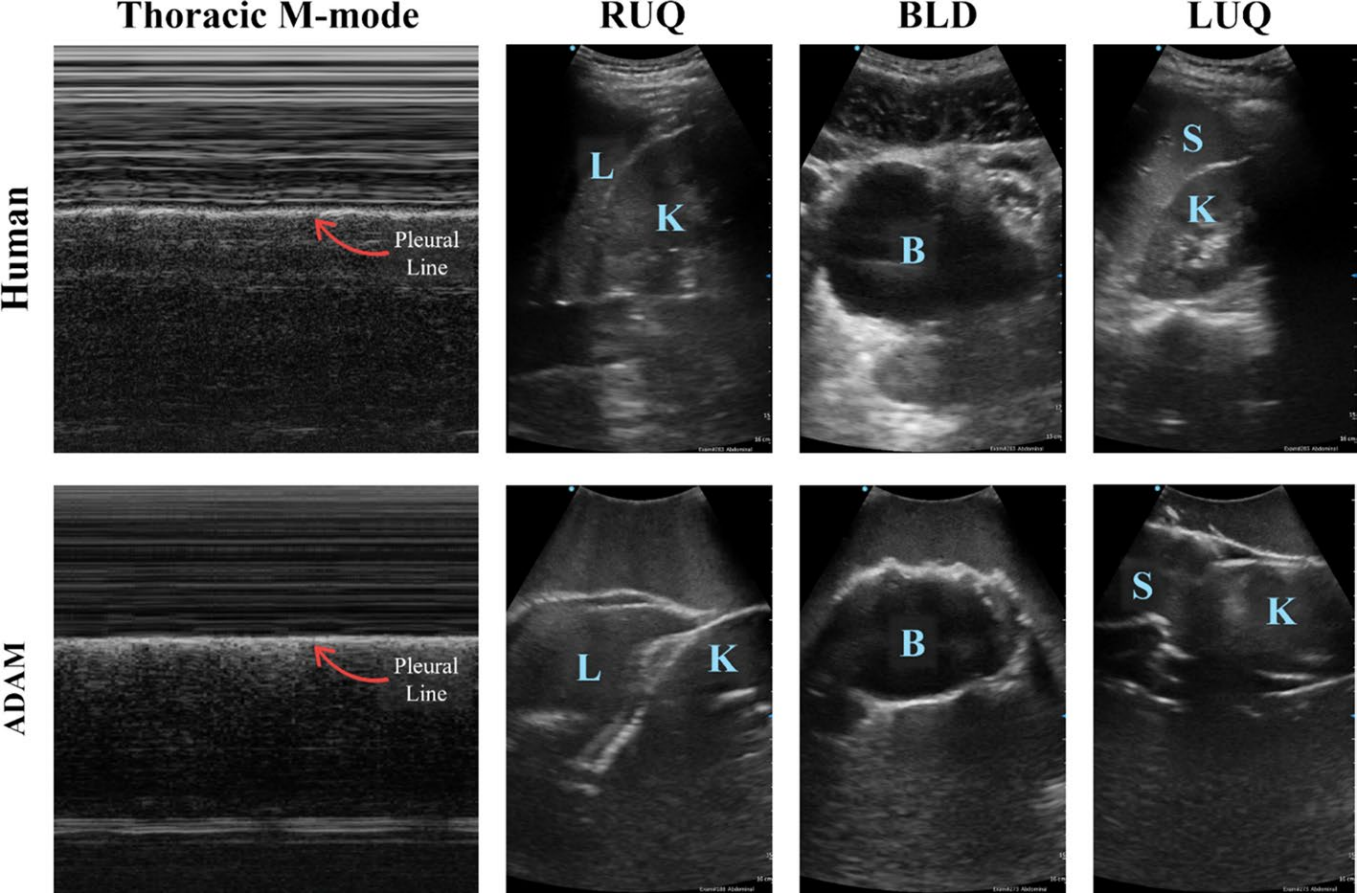
Comparison of US images obtained from a human and ADAM. Shown are representative images for each of the regions mentioned with labeled anatomical features (L = Liver, K = Kidney, B = Bladder, and S = Spleen). No human US images were used for AI model training and are only shown here for comparison

In a qualitative comparison, US images captured with the ADAM phantom without injuries were compared against human US images (Fig. 3). For thoracic images, pleural lines were similar in ADAM and human tissue as are the subcutaneous tissue regions of the image. Lung sliding effects on the M-mode image are similar between human and ADAM captured images. For abdominal scan sites, spleen, kidneys, bladder, and liver organs in each image are similar in size and orientation. However, tissue was more homogeneous overall in ADAM compared to human tissue based on the lower complexity of the gelatin substance surrounding the organs compared to human anatomy.

Anatomical guidance object detection models for the ribs, kidney, and bladder were successfully developed using YOLOv8 with training intersection-over-union (IOU) scores of 0.821, 0.798, and 0.906, respectively. However, when evaluating the object detection models with blind ADAM test phantoms, performance was slightly reduced with IOU scores of 0.648, 0.516, and 0.536 for the ribs, kidney, and bladder, respectively.

**Fig. 4:**
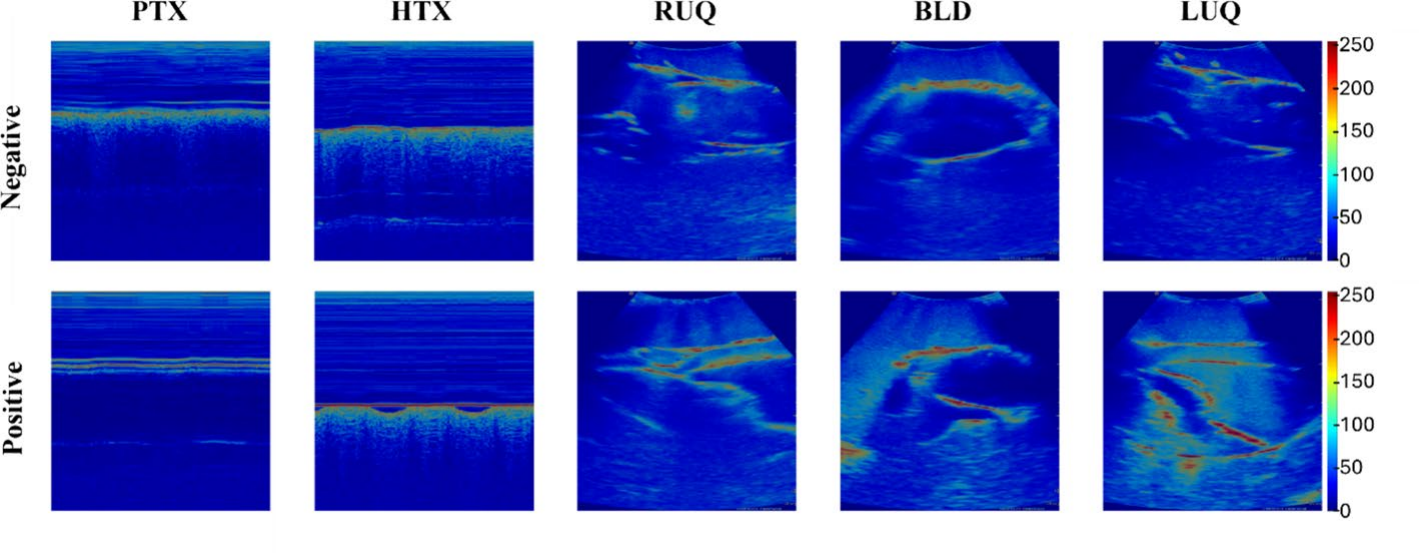
Representative ultrasound images for each injury condition at each scan point with GradCAM overlays. Negative states are shown in the *top row,* and the *bottom row* highlights representative positive injury conditions. For the thoracic site, PTX and HTX states are shown separately with two different negative states shown. GradCAM overlays are shown as heat maps, with red-yellow indicating regions where the the AI model focused on to make a decision while blue hues refer to regions of less importance to the AI model predictions

For diagnostic AI, overall performance metrics with blind test ADAMs for YOLOv8 and highest performing Bayesian optimized models are summarized in Table 1. YOLOv8 outperformed the Bayesian models for the thoracic with approximately 0.10 higher accuracy, but trailed behind the Bayesian model results for the other scan sites. Most notably were RUQ and BLD results where accuracy was 0.230 and 0.323 higher using the Bayesian model compared to YOLOv8, respectively. Gradient-weighted class activation mapping (GradCAM) overlays for positive and negative image types were generated for each scan site with the Bayesian Optimized model. GradCAMs identify “hot spots” which correlate with image regions most significant to the AI model and, for this use case, highlight the model's capability to identify key anatomical features in the case of RUQ, BLD, and LUQ scan sites (Fig. 4). The fluid accumulation site was identified as a “hot spot” in the image to exemplify how the AI model identified these anatomical relevant features when making model predictions. Similarly, for the M-mode image of the lungs, the AI model identified breathing signs in the negative image, fluid accumulation in the pleural space for HTX, and steady pleural line for the PTX site (Fig. 4), indicating successful model training for recognizing relevant anatomical patterns, given the resulting GradCAM overlays.

**Table 1:** Performance metrics in blind test phantom US scans for YOLOv8 and Bayesian optimized classification AI models; results are shown as mean results

Scan site	Accuracy	Precision	Recall	Specificity	F1 Score
YOLOv8					
Thoracic	0.817	0.916	0.817	0.860	0.862
RUQ	0.552	0.874	0.549	0.545	0.674
BLD	0.412	0.410	0.401	0.423	0.406
LUQ	0.711	0.781	0.656	0.745	0.731
Bayesian optimized					
Thoracic	0.715	0.594	0.993	0.528	0.737
RUQ	0.782	0.738	0.738	0.811	0.738
BLD	0.735	0.759	0.759	0.711	0.759
LUQ	0.772	0.576	0.576	0.969	0.576

## Discussion

Overall, the ADAM tissue phantom was successful at creating realistic US images and showed positive results in training AI models. In this work, we developed an ultrasound compatible thoracic phantom with modular injuries and the capability for simulated lung motion. The EVE system allowed for the collection of thoracic images and the simulation of PTX and HTX injuries, both of which were integral for training the AI models in this study.

The creation of the ADAM phantom involved the selection of ultrasound compatible materials that can withstand continuous force as would be experienced during examination. A lung phantom was successfully created with the ability to simulate realistic motion during ultrasound imaging and the added flexibility to adjust the breathing rate in real time. Although the current range of respiratory rates is quicker than typical physiology, this can be adjusted with higher torque, lower speed actuators. In addition to being able to include negative and positive injury states at each eFAST site, adjustable progression from a non-injured to a severe injured state is another feature of the ADAM phantom (Fig. 2). Injury severities are variable in clinical situations and can worsen with time, so this capability provides a useful training feature for using ADAM as an eFAST trainer. This is also important during the AI training process that can also benefit from subject and injury variability to reduce model overfit. Modular thoracic injuries were possible for both HTX and PTX as well as simulating fluid accumulation around kidneys, spleen and around bladder. As the abdominal organs can be reconfigured with each use, ADAM provides not only modular injury sizes but also slight subject variability.

To further highlight the utility of ADAM, we qualitatively compared US images captured at each ADAM scan location to representative human US scans (Fig. 3), and there was an overall similarity between images at each scan site. Similar breathing artifacts were evident in ADAM and human tissue for the thoracic site, and organs were similarly sized and oriented in the abdominal sites. However, ADAM cannot mimic the complex tissue layers and heterogeneous US properties found in human tissue which can be evident with the image comparison. This comparison to human images was qualitative and limited in scope as few human images were available at this time, and the images at each site are from a single healthy human subject. As such, this comparison cannot evaluate the similarities of ADAM injured states to human injuries. More human US image capture will be required to further validate the ADAM tissue phantom to human tissue.

A phantom development limitation was injury placement. For example, the injury sleeves would occasionally shift as the lung phantom moved inside the thoracic cavity. This limitation was not limited to the thoracic injuries but also the abdominal portion as well, where the organs would also become displaced if not seated correctly or if the gelatin solution was poured too quickly. More quantitative comparisons, such as measuring attenuation coefficients and overall echogenicity versus human tissue, will be considered in future work.

ADAM’s features were able to overcome the shortcomings of other commercially available phantoms. While phantoms such as the SonoSkin trainer are useful in teaching personnel how to conduct FAST and eFAST examinations, the lack of image variability is not suitable for training AI models. The Blue Phantom FAST exam trainer allows for real time scanning and simulation of hemorrhage; however, the imaging artifacts caused by fluid removal make the evaluation of negative scans unfeasible. The ability to alter injury severity, image lung motion along the entire thoracic region, and adjust respiratory rates are integral features for developing an AI triage tool that can be viable at the point of injury on a battlefield setting. The ADAM phantom may be used not only as an AI training tool, but also as a resource for medical personnel training. Tissue phantoms have been used to educate trainees in conducting eFAST examinations [16], due to their durability and the realistic US images they generate. During training scenarios, the ADAM phantom would be capable of producing image variance exposing trainees to varied injury severities, thus providing valuable triage experience.

In this study, we highlighted the application of the ADAM tissue phantom toward AI model development. The most important capability of ADAM for this task is to provide sufficient anatomical features along with subject variability to enable AI models to perform effectively on blind test subjects while relying on relevant anatomical features applicable to real-life scenarios. The object detection guidance models performed well during training and maintained blind performance higher than a 0.50 IOU performance threshold. However, there was a noticeable drop in performance between training and blind testing, indicating the models may be overfit to the training data. Therefore, greater subject variability or enhanced image augmentation may be required to improve anatomical guidance model performance. Successful anatomical guidance models with a bounding box output can aid in real-time US scans to confirm to the end user that the US probe is properly positioned at the scan site, lowering the skill threshold for this triage examination if properly implemented.

In addition, diagnostic models were developed with mixed success. YOLOv8 models were initially developed; however, performance was insufficient during blind testing due to overfitting issues for most of the abdominal scan sites. Instead, we highlighted how the phantom could be used to tune Bayesian optimization parameters to customize model performance for this specific use case. All scan site blind tests exceeded 70% accuracy, and GradCAM overlays (Fig. 4) illustrated that AI models identified proper anatomical injury landmarks, a critical design feature required for the developed phantom to be suitable for this AI application. If the AI models were tracking image artifacts or anatomically irrelevant features to make model predictions, the AI training benefit of ADAM would be reduced, and a larger quantity of human images would be needed. In future work, greater subject variability and injury severity magnitudes are still needed to further improve AI training and evaluate AI model sensitivity. Finally, comparing the training results against human-trained cases to demonstrate how the AI focused on similar regions of interest to identify injuries supports the future development of the tissue phantom presented here. Additional comparison to human images will determine overall model generalizability to predict accurately for both human and phantom tissues datasets.

## Conclusion

To overcome some of the challenges posed by limited trained personnel and the difficulties of US imaging in battlefield settings, the ADAM phantom was developed. This high-functioning tissue phantom was designed to simulate multiple thoracic and abdominal injuries that are routinely assessed during eFAST exams. It incorporates realistic anatomy and simulation of injury to support anatomical guidance and injury diagnostic AI models to determine injury status and severity. Additionally, the injury diagnostic models showed promise in their ability to evaluate injury status using relevant anatomical features. Model performance may be improved through further refinement and optimization with approaches that focus on model generalization. Overall, the ADAM phantom has the potential to serve as an effective subject for developing and testing AI models for US image interpretation and overcoming the challenges faced in the deployment of ultrasound imaging techniques in combat casualty care settings.

## Materials and methods

### Tissue phantom development

**Fig. 5:**
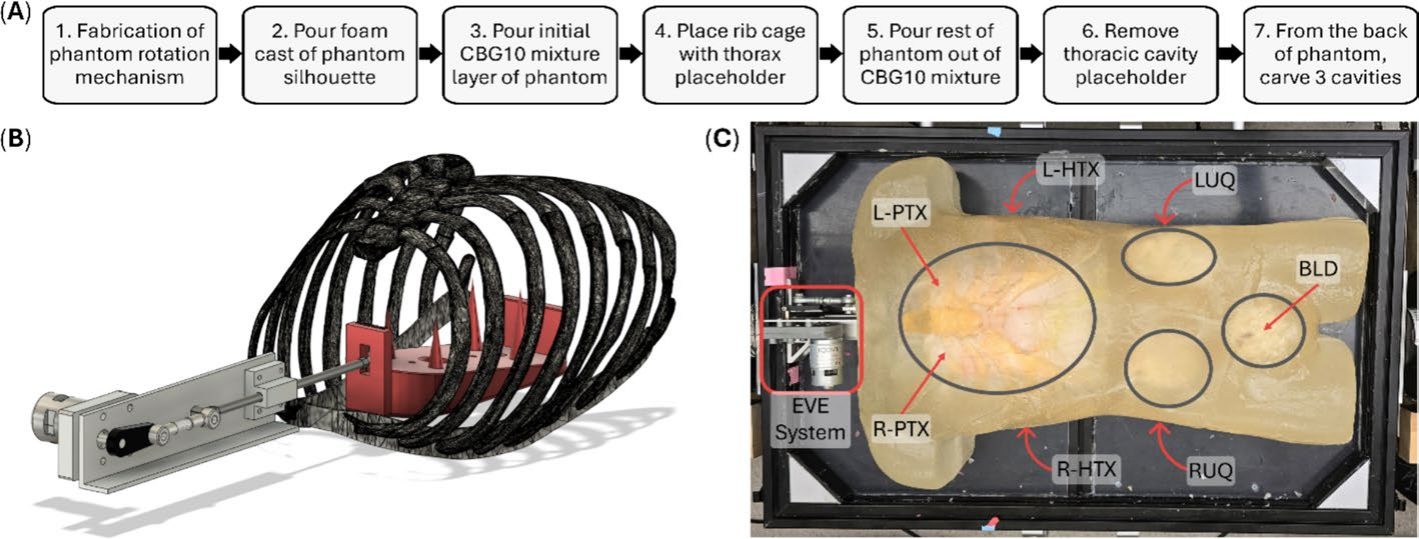
Making of ADAM tissue phantom for simulating eFAST scan points. **A** Step-by-step description of the phantom making process. **B** Computer design of the EVE system for modeling lung motion. **C** Picture of complete tissue phantom with identified cavities (*grey ovals*), breathing apparatus (EVE system, *red square*), and approximate locations of scan points for imaging (*red arrows*)

A full-sized human torso tissue mimicking phantom was created to collect eFAST images for guidance and diagnostic AI model training. The tissue phantom development was done in steps (Fig. 5A) to minimize bubbles and ensure the rib cage was placed correctly. The phantom model was developed based on a previous eFAST model [15] with some functional improvements: (1) a new thoracic breathing mechanism, (2) fluid injuries at each site, (3) modular injury creation, and (4) an inversion mold. The last improvement was meant to assist with the handling and preservation of the tissue phantom, as a rotating mechanism to invert the tissue phantom during pouring and imaging processes. This was created by attaching the phantom mold to a wooden table containing a cutout large enough to fit the mold and allow tolerance while rotating. The rotating mechanism was driven by attaching two motor-driven gears to the mold. Once rotated, the mold was securely anchored using linear actuators.

The phantom was created by pouring 10% clear ballistic gel (CBG10; Clear Ballistics, Greenville, South Carolina, USA) containing 1% w/w silica gel (Sigma-Aldrich, St. Louis, Missouri, USA). The silica gel acts as an ultrasound scattering agent which mimics heterogeneity of real tissues, which helps the ultrasound waves scatter and attenuate [17]. When pouring the CBG10, a 3D printed ribcage made from High Temperature Resin (FormLabs, Somerville, Massachusetts, USA), with a printed temporary lung mold embedded within the rib cage. Once the phantom was fully poured and cooled, the back of the phantom was carefully carved and the temporary mold was taken out, leaving a hollow rib cavity. Three more cavities were made for the right upper quadrant (RUQ), left upper quadrant (LUQ), and pelvis (BLD) at their corresponding anatomical sites. Internal organs were created by pouring the same ballistic gel and silica gel mixture into molds for kidneys, liver, and spleen 15. Bladder and rectum were poured using CBG10.

We chose CBG10 as the main material for casting the phantom due to its durability, since the thoracic phantom must withstand continuous forces exerted during an ultrasound examination. Other materials were preliminarily tested to create a phantom and produce more physiologically accurate ultrasound images. CBG20 was experimented with and was found to be too stiff and prevented the lung motion motor from working correctly. The EVE system applies tensile and compressive forces as it moves through the torso and simulates breathing. This mechanical stress is further compounded by the addition of the modular injuries. We tested various mixtures of CBG with other types of ultrasound compliant materials, then proceeded to tensile test on a uniaxial test platform. Stress–strain curves were generated, with pure CBG10 presenting the most favorable behavior. To simulate breathing, we created a linear motion mechanism that slides a foam lung model across the ribcage. The simulated lung tissue was created by pouring Soma Foama™ 25 (Smooth-On Inc, Macungie, Pennsylvania, USA) into the hollow ribcage cavity. Once cured, the foam lung was removed and shaved to allow the lung to slide inside the cavity against the cavity walls. The foam lung was then attached to a 3D printed cart and sled along a T-rail using a 160 reps per minute reciprocating linear actuator (Amazon, Seattle, WA, USA), as seen in Fig. 5B. Breathing rates were controlled by using a DC motor speed regulator to control the actuator.

For abdominal scan sites, the corresponding organs were placed to match anatomical relevance (Fig. 5C). Similar to the previous phantom model [15], the cavity was then filled with a 10% gelatin mixture made with a 2:1 ratio of water and evaporated milk with flour at a 1% w/v concentration. The gelatin was poured in multiple layers to avoid displacement of the organs. The phantom was then placed at 4 °C to allow the gelatin to solidify overnight. Ultrasound compatible sleeve covers were placed in the interface between the organs to create modular injuries. The sleeve covers were converted into bags by vacuum sealing the ends, and tube fittings were added to these pockets which allowed for a syringe to be connected to ensure precise control over the amount of water or air added. This simulated free fluid in between the organs, similar to real injuries seen in positive eFAST scans [18]. Full AH injuries were created by inserting 40 mL of water in the injury pockets. Full HTX and PTX were created by inserting 40 mL of water and 80 mL of air, respectively. By controlling the amount of fluid or air, injury severity was modulated to simulate injury progression. This feature enabled the creation of varied injury levels, ideal for training AI models to not only detect the presence of an injury but also assess the severity of the injury from ultrasound scans.

### Image capture and processing

Two types of AI models were developed using US images: anatomical guidance and injury diagnostic. These models were trained using a dataset of frames extracted from 10 second US video clips captured using a Vscan Air (GE Healthcare, Chicago, IL). For anatomical guidance model training, two sets of US B-mode videos were collected: (1) medial to lateral and (2) distal to proximal scan swipes at each site. For abdominal scan sites, the curvilinear probe (2–5 MHz) was used for video capture aimed at keeping anatomical landmarks of interest in view, namely bladder and kidneys. For thoracic scan sites, the linear probe (3–12 MHz) was guided along the ribs, starting from the upper chest and moving across the armpit to the lower ribs. Sets of images were captured for both negative and positive injury. Once collected, data was prepared by labeling the following anatomical landmarks with bounding boxes: kidney for the RUQ and LUQ views, bladder for BLD view, and ribs for the thoracic views. To achieve this, US clips were cropped to remove the US system user interface, converted to grayscale and separated into individual frames before being labeled using video annotation software, such as COCO annotator and MATLAB toolboxes.

For injury diagnosis models, three B-mode US videos were collected at each scan site. For abdominal scan sites, these videos imaged the anatomical features of interest while the probe was slowly tilted to capture views from different angles. For thoracic scan sites, the probe was placed at intercostal sites, ensuring that two ribs and the inner pleural space were in view. These US videos were captured for both negative and positive injury states and then used as training data. To introduce subject variability, injury diagnostic and anatomical guidance US images were captured on three ADAM iterations. Each iteration of ADAM was made by repositioning the organs and then repouring the gelatin mixture so that anatomical variability could be present within the collected images.

For qualitative comparison of ADAM to human anatomy, US images were captured at each eFAST scan site (thoracic, RUQ, LUQ, and BLD) in human volunteers who provided verbal consent. All US image capture followed the probe and image capture setup for ADAM using the VScan Air US system. For thoracic image capture, M-mode captures were recorded only. For abdominal image capture, B-mode US videos were captured at each scan site. Representative frames were extracted from video files for comparison to ADAM. No human US images were used for AI model training.

### AI model training

The processed US images described above were used to train AI models for each scan site using two approaches. First, object detection models for anatomical guidance were developed for anatomical detection of ribs, kidneys, and bladder features at the respective scan points using a YOLOv8 model architecture [19] which demonstrated previous success in US applications [12] utilizing the YOLOv8-s pretrained object detection weights. Next, classification models using the same YOLO framework utilizing up to five different ADAM iterations were used for training injury diagnostic models while the data from three separate phantoms were blind subjects used to evaluate model performance. In total, training data for anatomical guidance models varied between scan sites with 2200–3800 images for training with an extra 550–850 images for validation data. The abdominal injury diagnostic model training data contained 2800–3000 images with about 300 images separated for validation, and thoracic injury diagnostic models were trained off a much smaller dataset of 368 images with just 30 M-mode images used for validation. Using previously developed methods [12], M-mode images were created from a moving window size of 5 s with a 0.5 s sliding window of B-mode captures of the thoracic cavity by identifying a midpoint between the ribs as identified by the anatomical guidance AI model.

The data separated for YOLOv8 validation were blind videos files but using the same ADAM phantom iteration found in the training data. However, validation videos contained a view of the ADAM anatomy that varied from what was present in the training data. Therefore, despite using the same ADAM iteration in both training and validation data, there was still variance between both datasets to minimize overfitting at each ADAM scan site. Accuracy metrics were collected using test data collected on ADAM phantom iterations completely blind to either the training or validation data.

Each scan site had binary model types (positive or negative for injury) except for the lung model which was three class (HTX, PTX, or negative). For each YOLOv8 model the following default training settings were used: 100 training epochs, a batch size of 16, a learning rate of 0.1, a weight decay of 0.0005, and no early stopping. Default YOLOv8 image augmentations were also applied to a fraction of the training data, including adjusting the hue, saturation and brightness of the images, translating the image vertically or horizontally by a factor of 0.1 of the image size, scaling the image by a gain factor of 0.5, flipping the image by the vertical axis, and erasing part of the image so the model is able to recognize less obvious features in the image.

Each of the conventional YOLOv8 injury diagnostic models’ performance was then compared to a classification model whose architecture was developed utilizing a Bayesian optimization process. The Bayesian optimization approach used a series of convolutional layers with ReLU activators and max pooling layers followed by a fully connected layer, dropout layer, and final classification layer. The number of convolutional neural network layers, number of nodes, filter size, dropout amount, and size of fully connected layer were all hyperparameters, as were affine augmentation types with a variable extent of turning for each augmentation as additional tunable parameters. Lastly, the batch size and learning rate were set as hyperparameters in the optimization setup. The same training dataset for YOLOv8 training was used for Bayesian optimization with the exception that data captured from an additional blind ADAM phantom iteration was used for validation with a validation loss patience set to 5 epochs for a maximum of 20 epochs. Furthermore, an additional blind ADAM phantom iteration was used as blind test data during Bayesian optimization to evaluate each iterations performance where the goal of the 100-iteration optimization problem was to maximize blind test accuracy. The top 5 model configurations were trained in triplicate for 100 epochs with a validation loss set to 5 epochs. A final blind ADAM phantom iteration not used during Bayesian optimization was used for assessing overall testing performance on each optimized model. These US scans were further used to construct GradCAMs to highlight regions of the US scan where AI decisions were focused to bolster explainability of the AI model results.

## Data Availability

The data presented in this study are not publicly available because they have been collected and maintained in a government-controlled database located at the U.S. Army Institute of Surgical Research. This data can be made available through the development of a Cooperative Research and Development Agreement (CRADA) with the corresponding author.

## Declarations

### DoD disclaimer

The views expressed in this article are those of the authors and do not reflect the official policy or position of the U.S. Army Medical Department, Department of the Army, DoD, or the U.S. Government.
